# Shaping of Monocyte-Derived Dendritic Cell Development and Function by Environmental Factors in Rheumatoid Arthritis

**DOI:** 10.3390/ijms222413670

**Published:** 2021-12-20

**Authors:** Frédéric Coutant

**Affiliations:** 1 Immunogenomics and Inflammation Research Team, University Claude Bernard Lyon 1, Edouard Herriot Hospital, 69003 Lyon, France; frederic.coutant@univ-lyon1.fr; 2 Immunology Department, Lyon-Sud Hospital, Hospices Civils of Lyon, 69310 Pierre-Bénite, France

**Keywords:** human dendritic cells, rheumatoid arthritis, aryl hydrocarbon receptor, ACPA, biotherapies, infliximab, tocilizumab, abatacept, tofacitinib

## Abstract

Dendritic cells (DC) are heterogeneous cell populations essential for both inducing immunity and maintaining immune tolerance. Chronic inflammatory contexts, such as found in rheumatoid arthritis (RA), severely affect the distribution and the function of DC, contributing to defective tolerance and fueling inflammation. In RA, the synovial fluid of patients is enriched by a subset of DC that derive from monocytes (Mo-DC), which promote deleterious Th17 responses. The characterization of environmental factors in the joint that impact on the development and the fate of human Mo-DC is therefore of great importance in RA. When monocytes leave the blood and infiltrate inflamed synovial tissues, the process of differentiation into Mo-DC can be influenced by interactions with soluble factors such as cytokines, local acidosis and dysregulated synoviocytes. Other molecular factors, such as the citrullination process, can also enhance osteoclast differentiation from Mo-DC, favoring bone damages in RA. Conversely, biotherapies used to control inflammation in RA, modulate also the process of monocyte differentiation into DC. The identification of the environmental mediators that control the differentiation of Mo-DC, as well as the underlying molecular signaling pathways, could constitute a major breakthrough for the development of new therapies in RA.

## 1. Introduction

Originally defined by Ralph Steinman on the basis of their unique morphology that distinguished them from macrophages, dendritic cells (DC) are antigen-presenting cells, best known for the potent and unique abilities to initiate adaptive immunity against infectious agents and cancer cells [1]. However, DC are not only a crucial line of defense against invading pathogens, they are also key regulators of the balance between immunity and tolerance, thus actively participating in the maintenance of tissue homeostasis by dampening uncontrolled inflammation [2,3]. In view of all these functional properties, any disruption of the subtle homeostatic equilibrium in the DC system can lead to the development of chronic inflammatory/autoimmune diseases [4].

Human DC are a heterogeneous population that have been classified into distinct subsets, according to ontogeny, phenotype and transcriptional profile [5]. All the subsets of DC have in common their ability to take up antigens, present them to antigen-specific naive T cells, and promote coordinated adaptive responses. The DC family comprises four major subsets: the type 1 and type 2 classical DC (cDC1 and cDC2 respectively), the plasmacytoid DC (pDC), and the monocyte-derived DC (Mo-DC). Additionally, two distinct DC subsets have also recently been described: the transitional DC, which display an intermediate phenotype between cDC and pDC, and the DC3, which present a phenotype close to but distinct from Mo-DC [5,6]. Functionally, cDC are more specialized in T-cell interactions, whereas pDC are well known for their ability to rapidly produce large quantities of type I interferon upon viral encounter. A panel of traditional and recently described markers are currently used to characterize these DC subsets, such as CD141 and C-type lectin domain family 9 (CLEC9A) for cDC1; CD1c and CLEC10A for cDC2; CD123, CD303 and CD304 for pDC; CD1c and CD163 for DC3; AXL and SIGLEC6 for transitional DC [7].

The term Mo-DC refers to a subset of DC derived from monocytes that differentiated into DC after having infiltrated a tissue in an inflammatory context. However, Mo-DC have also been described in steady-state conditions [8,9]. In humans, Mo-DC have been described in several inflammatory contexts, such as infection, cancer, allergy and chronic inflammatory diseases. Mo-DC are increased significantly during tuberculosis infection, especially in pleural effusions, and they have also been identified in leprosy lesions [10,11]. Mo-DC have also been detected in malignant pleural effusions of non-small-cell lung cancers, as well as in ascites from untreated ovary and breast cancers [12,13]. They have also been recently identified in the context of chronic inflammatory diseases, such as rheumatoid arthritis (RA). The Mo-DC phenotype is complex. These cells express cDC2 markers (CD1c and FcεRI), in-vitro-differentiated Mo-DC markers (CD1a), monocyte/macrophage markers (CD14, CD206), as well as the surface lymphocyte-specific protein 1 (LSP-1) [13,14]. Interestingly, the development of an in-vitro culture model to generate Mo-DC revealed that these cells express also a large and atypical panel of C-type lectin receptors, including isoforms of CD209 and CD206, CD303 and CD207, as well as intracellular proteins at their surfaces, such as the lysosomal protein CD208 [15]. From a functional point of view, Mo-DC exhibit high plasticity, with the ability to induce Th1 responses in cancer, Th2 in allergic contexts, and Th17 in chronic inflammatory situations such as RA [12,13,16].

**Figure 1:**
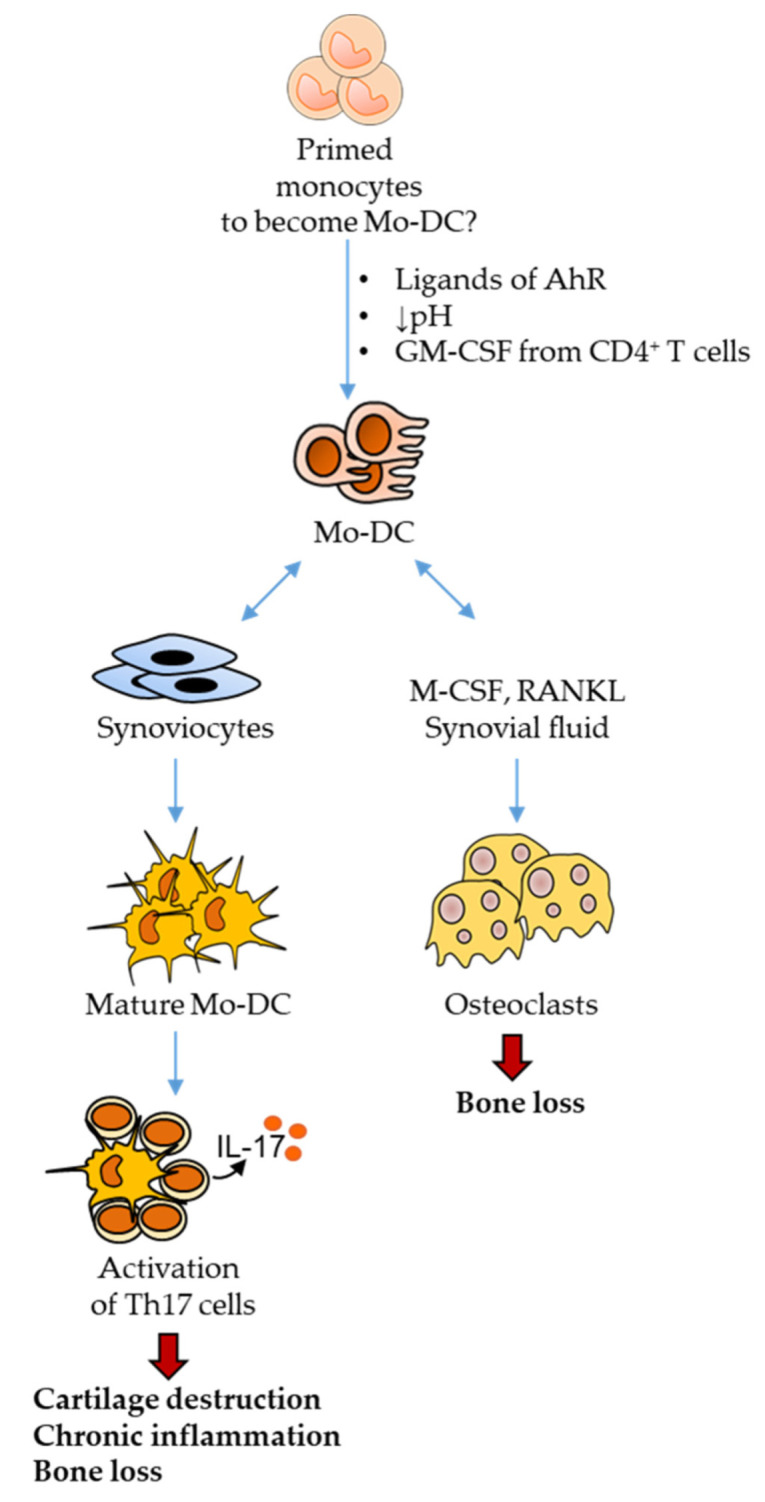
Regulation of monocyte-derived dendritic cells differentiation and function by environmental factors in rheumatoid arthritis. Several environmental factors influence the differentiation of Mo-DC, such as the natural ligands of the aryl hydrocarbon receptor, the extracellular acidosis, or the GM-CSF produced by synovial CD4^+^ T cells. Interactions with synoviocytes can also affect the differentiation of Mo-DC as well as their maturation. Whether the program of monocyte differentiation into Mo-DC is already initiated within the monocyte, before its penetration into the synovial tissue is still unclear. Activated Mo-DC are potent inducers of Th17 cells that participate to cartilage destruction and chronic inflammation through the secretion of IL-17. Mo-DC can also transdifferentiate into osteoclasts, thus promoting bone loss. Abbreviations: AhR: Aryl hydrocarbon Receptor; GM-CSF: Granulocyte-Macrophage Colony-Stimulating Factor; M-CSF: Macrophage Colony-Stimulating Factor; Mo-DC: Monocyte-derived Dendritic Cells; RANKL: Receptor Activator of Nuclear factor Kappa-B Ligand.

An intriguing and still incompletely resolved question is the nature of the cellular and molecular factors that govern the differentiation of monocytes into Mo-DC. The identification of such factors is particularly important in the context of chronic inflammatory diseases such as RA, in the hope of impacting on the dysregulations of this subset of DC. In this review, after a brief description of the potential implications of Mo-DC in the in the RA pathogenesis, we will focus on environmental factors which are able to affect the differentiation and the fate of Mo-DC, and we will discuss their relevance in the context of RA (Figure 1). The discussion is limited to recent advances in the field of human Mo-DC.

## 2. Role of Monocyte-Derived Dendritic Cells in the Pathogenesis of Rheumatoid Arthritis

In 2013, CD14^+^CD1a^+^CD1c^+^ DC were identified in the synovial fluid of patients with RA [13]. When cultured with naive CD4^+^ T cells, these Mo-DC specifically induce the production of IL-17 by T cells through the secretion of TGFβ, IL-1β, IL-6, and IL-23. It is, however, important to note that these ex-vivo Mo-DC do not spontaneously secrete IL-23, a key cytokine for maintenance/expansion of Th17 cells, and they need to be stimulated with a TLR2 ligand to achieve this. In contrast, ex-vivo macrophages do not secrete IL-23, even after stimulation with a TLR2 ligand. The activation of Th17 by Mo-DC cells may contribute to the pathogenesis of RA in two main ways. First, IL-17 may perpetuate and amplify local inflammation by acting in synergy with the TNFα. Indeed, IL-17 synergizes with TNFα on synoviocytes to induce the expression of proinflammatory effector molecules, such as IL-6 and IL-8 [17]. The combination of these two cytokines impact also on endothelial cells, by favoring a pro-inflammatory, pro-coagulant and pro-thrombotic phenotype [18]. Second, IL-17 participates in the structural degradation of joints by activating chondrocytes and inducing the production of enzymes that degrade the cartilage. IL-17 contributes also to bone destruction through the dysregulation of the receptor activator of nuclear factor κ-B (RANK)-RANK ligand (RANKL) signaling pathway, which is critical for the differentiation and the survival of osteoclasts. In addition, recent studies have unveiled a new pathogenic axis in RA, involving Mo-DC, Th17 cells, and autoreactive plasma cells [19]. When activated, Th17 cells not only produce IL-17, but they also produce IL-21 and IL-22. These cytokines can down regulate the expression of the enzyme sialyltransferase, ST6GAL1, which is normally expressed by plasma cells. This enzyme is involved in the addition of residues of sialic acids on the glycosylated chains present on antibodies such as anti-citrullinated protein antibodies (ACPA), the highly specific autoantibodies of RA. By down regulating the expression of ST6GAL1 in autoreactive plasma cells, IL-21 and IL-22 cause a shift from normally glycosylated non-pathogenic ACPA toward inadequately glycosylated ACPA with pro-osteoclastogenic activities [20,21].

The nature of the environmental factors and the regulatory mechanisms that govern the differentiation of monocytes that infiltrate inflammatory tissues such as the synovial tissues in RA, are poorly understood. Upon entry into the tissues, monocytes interact with a myriad of environmental stimuli influencing their differentiation. For instance, IL-6 from fibroblasts promotes monocyte differentiation to macrophages, whereas TNF skews the monocyte differentiation from macrophages to DC [22,23]. Pro-and anti-inflammatory lipids affect also the monocyte-to-DC transition [24,25]. Numerous environmental stimuli have thus been described, using in-vitro culture models of Mo-DC generated with GM-CSF and IL-4. We will only review, here, the environmental factors described recently in cell culture models relevant for the generation of Mo-DC close to ex-vivo Mo-DC or relevant in the context of RA.

## 3. Environmental Factors in Rheumatoid Arthritis That Govern the Monocyte Differentiation into Dendritic Cells

Among the many environmental factors described as being able to affect the generation of Mo-DC, four of them appear to be particularly relevant in the context of RA: (i) the dietary metabolites that are agonists of the aryl hydrocarbon receptor (AhR), (ii) the extracellular acidosis, (iii) the GM-CSF produced by synovial CD4^+^ T-cells, and (iv) the synoviocytes and the synovial fluid.

### 3.1. Control of the Monocyte-to-DC Transition by the Ligands of the Aryl Hydrocarbon Receptor

The AhR is a cytoplasmic ligand-activated transcription factor that exhibit immunoregulatory functions once activated [26]. The activation of the AhR has been shown to skew the monocyte differentiation to Mo-DC, when monocytes are simultaneously exposed to AhR ligands, M-CSF, IL-4 and TNFα. The Mo-DC generated under these conditions exhibit a phenotype close to that of the Mo-DC found in inflammatory pathological contexts. Natural ligands of AhR ligands are present in the diet, as natural compounds in food, and are also generated by the metabolism of the microbiota, particularly by the tryptophan catabolism. Interestingly, the activation of the AhR promotes also the differentiation of Th17 cells and consequently the production of the Th17-related cytokines IL-17 and IL-22 [27]. Activation of AhR could, therefore, play a key role in the pathogenesis of RA, by two mechanisms. First, by switching monocyte differentiation towards Mo-DC that are particularly effective in inducing Th17 responses, but also by promoting directly the differentiation of Th17 cells. Moreover, expression of AhR has been observed within the synovial tissues of patients with RA. Importantly, evidence of AhR activation, indicated by CYP1A1 and AHRR gene expression, was found only in the synovia of patients who smoked [28]. This is particularly relevant in RA since environmental pollutants and cigarette smoke significantly increase the risk of RA, and both of them contain ligands of AhR [29]. Collectively, these data suggest a role for environmental ligands of AhR that may contribute to RA disease severity by affecting the monocyte to Mo-DC transition.

### 3.2. Impact of the Extracellular Acidosis on the Monocyte-to-DC Transition

The maintenance of intracellular and extracellular fluid pH is critical for maintaining cellular biochemical reactions and tissue homeostasis. Although the human body has elaborated a variety of mechanisms to control the pH, local and even systemic pH fluctuations occur frequently under pathological conditions. Local acidification may develop during chronic inflammation, notably in the joints of patients with RA. The pH of the synovial fluid of patients with RA may reach values of 6.8 to 7.1, whereas the pH of the synovial fluid of healthy individuals ranges from 7.4 to 7.8 [30,31,32]. Obviously, acidic microenvironments have an impact on the function of stromal cells in the joints, but also on immune cells that infiltrate the inflammatory tissue. The impact of extracellular acidosis on human dendritic cell function has been well studied, and it induces the maturation of DC generated from monocytes in the presence of GM-CSF and IL-4 [33]. When these cells are exposed to transient acidic conditions, they acquire a mature phenotype with an up-regulation of MHC class II molecules and the costimulatory molecules CD40 and CD86, as well as the ability to stimulate the secretion of IFNγ by CD4^+^ T cells, via the secretion of IL-12p70. By using the culture model of Mo-DC generated from monocytes incubated with M-CSF/TNFα/IL-4, which yields a heterogeneous population of cells including Mo-DC closer to the Mo-DC found in inflammatory fluids of patients, Diaz et al. have found that low pH markedly promotes the differentiation of human monocytes into Mo-DC [34]. In these culture conditions and at pH 6.5, most of the cells differentiated into Mo-DC, whereas cells cultured at pH 7.3 differentiated into macrophages. At pH 7.3, the addition of AhR ligands in the culture medium skew the differentiation of monocytes from macrophages to Mo-DC, highlighting the important role of AhR in the process of differentiation of Mo-DC. Mo-DC generated at pH 6.5 stimulate also the production of IFNγ by CD4^+^ T cells. The molecular mechanism induced by low pH and allowing the differentiation of Mo-DC involves the inhibition of the cellular nutrient sensor named mammalian target of rapamycin (mTOR)C1.

In conclusion, extracellular acidosis as observed in joints of patients with RA, could constitute a key environmental factor that locally promotes the differentiation of monocytes into Mo-DC.

### 3.3. Control of the Monocyte-to-DC Transition by the GM-CSF Produced by CD4^+^ T Cells

In RA, the synovial tissue is also infiltrated by T cells that participate in the activation of stromal cells and immune cells, transforming them into tissue-destructive effector cells. The presence in the synovial tissue of aggregates of T and B cells, infiltrated by DC and called ectopic lymphoid structures, provides compelling evidence of close interactions between DC and/or differentiating DC and T cells in moderate to severe RA [35,36]. CD4^+^ T cells are a recognized source of GM-CSF [37]. In human, GM-CSF has been reported to be produced by T helper 1 cells, but also by a distinct subset of T cells, known as GM-CSF-producing T helper cells [38,39].

In RA, CD4^+^ T cells are also an important source of GM-CSF, and GM-CSF production by these cells is significantly enhanced by IL-12 and IL-15 [40]. Interestingly, CD4^+^ T cells isolated from the synovial fluid of patients with RA and cultured in the presence of allogeneic monocytes promote the transition of monocytes to Mo-DC. This effect is mediated by the GM-CSF produced by CD4^+^ T cells, and Mo-DC generated in this culture system promoted Th17 and Th1 responses in mixed leukocyte reactions. Although this interesting in-vitro model allows the generation of Mo-DC close to those observed in the synovial fluid of patients with RA, it cannot fully recapitulate the Mo-DC generation that takes place in vivo. In particular, the Mo-DC thus generated do not express the FcεR1, which is present on the surface of the Mo-DC found in the synovial fluid of patients with RA, suggesting that other factors than GM-CSF contribute, in vivo, to the generation of Mo-DC.

### 3.4. Impact of the Synoviocytes on the Monocyte-to-DC Transition

When monocytes exit the blood and infiltrate the synovial tissue, they interact with plethora of soluble molecules, and also with the local dysregulated synoviocytes. Two in-vitro culture models were recently developed in order to recapitulate the synovial microenvironment and to analyze its impact on the differentiation process of Mo-DC and on the function of Mo-DC.

The first model relies on the use of conditioned media from ex-vivo synovial tissue biopsies of patients with RA. When added to Mo-DC differentiated with GM-CSF and IL-4 for 7 days, this explant-conditioned medium induces an upregulation of the maturation marker CD83 at the surface of Mo-DC, and the release of pro-inflammatory cytokines and chemokines by Mo-DC [41]. Mo-DC exposed to the explant-conditioned medium have metabolic alterations with an increased expression of glycolytic genes, which illustrates the importance of metabolic regulation in the control of Mo-DC function. The induction of the maturation program by unknown soluble components released in the explant-conditioned medium is at least partially dependent of the activation of the signal transducer and activator of transcription 3 (STAT3).

The second in-vitro culture model is based on the effect of synoviocytes from patients with RA on monocyte-to-DC transition [15]. In this model, monocytes are cocultured with RA synoviocytes over 5 to 7 days. This model demonstrated that RA synoviocytes, alone, do not support the differentiation of monocytes into Mo-DC. Even more surprisingly is the analysis of cells obtained after coculture of monocytes with RA synoviocytes in the presence of the cocktail of differentiation described by Goudot et al. [11]. After 5 days of incubation in these conditions, only 40% of cells acquired a phenotype compatible with Mo-DC, although most of the specific markers (CD209-like, CD206-like, CD1a) were more weakly expressed by these cells, compared with Mo-DC generated by culturing monocytes in the presence of the cocktail, without RA synoviocytes. These coculture experiments clearly demonstrate that synoviocytes from patients with RA are not able to induce the full differentiation of monocytes into Mo-DC. It could also explain why Mo-DC are detected in the synovial fluid of patients with RA, while no study has reported their presence in the synovial tissue, in close contact with RA synoviocytes. A possible hypothesis would be that the program of monocyte differentiation into Mo-DC is already initiated within the monocyte, before it exits the blood and enters into the synovial tissue, as recently suggested [42]. The contact of differentiating Mo-DC with dysregulated RA synoviocytes and soluble molecules released by these cells could then constitute a decisive trigger which will determine the functional and migratory properties of these cells. Some of these Mo-DC could migrate within particular structures found in the synovial tissue of RA patients, the ectopic lymphoid-like structures, while others migrate to lymph nodes or into the synovial fluid, thus acquiring a distinct phenotype.

In conclusion, exciting research is still needed to elucidate the mechanisms that lead to monocyte-to-DC transition in RA, and to define the precise role of synoviocytes.

## 4. Regulation of Monocyte-Derived Dendritic Cell Differentiation into Osteoclast

Increased osteoclastogenesis is an early pathogenic feature of RA. Interestingly, there is increasing evidence that Mo-DC, in some inflammatory pathological conditions, can transdifferentiate toward potent osteoclast (OC) precursors [43]. This was initially demonstrated by Rivollier et al. in 2004, by using Mo-DC generated in vitro with GM-CSF and IL-4 [44]. By exposing immature Mo-DC to M-CSF and RANKL for 6 days, they found that a transdifferentiation program operated through the fusion of immature Mo-DC. Importantly, the authors reported that this process was greatly enhanced by adding synovial fluid from patients with RA and involved both proinflammatory cytokines and components of the extracellular matrix, such as hyaluronic acid.

Fifteen years later, after the description of the Mo-DC/OC transdifferentiation, Krishnamurthy et al. demonstrated that this mechanism was dependent of protein citrullination by peptidyl arginine deiminase (PAD) enzymes [45]. PAD enzymes govern the citrullination process, a post-translational conversion of arginine to citrulline residues, which is dysregulated in RA and drive the production and maintenance of ACPA. Interestingly, the authors found that purified ACPA induce the Mo-DC/OC transdifferentiation, probably by targeting citrullinated actin and vimentin expressed at the surface of immature Mo-DC.

Taken together, these results provide two key insights into the physiology of Mo-DC. First of all, they illustrate the great plasticity of these cells. Then, they provide additional elements suggesting a potentially pathogenic role of ACPA in RA, via their impact on Mo-DC. This had already been suggested by recent work highlighting the importance of the glycosylation of ACPA on their pathogenic functions, and the ability of Mo-DC to regulate the glycosylation process [19].

## 5. Impact of Targeted Therapies on Monocyte Differentiation into Dendritic Cells

The repertoire of therapeutic drugs with benefit in the treatment of RA has grown steadily, in particular with the emergence of the targeted synthetic and biologic disease-modifying anti-rheumatic drugs (DMARD). Biologic DMARD have four underlying modes of action: (1) the neutralization of either TNFα or the TNFα receptor, (2) the neutralization of IL-6 directly or the blockage of the IL-6 receptor, (3) the negative regulation of the co-stimulation process between antigen presenting cells and T cells, and (4) the depletion of B cells. Targeted synthetic DMARD correspond to small molecules targeting intracellular transduction pathways involved in the cytokine-mediated induction of inflammatory responses, namely the JAK-STAT pathway. Several works have illustrated the impact of DMARD on Mo-DC.

Therapeutic blockade of TNFα with neutralizing antibodies (e.g., infliximab) or soluble TNFα receptor immunoglobulin constructs (e.g., etanercept) has been the gateway for the biologic DMARD in the treatment of RA. Considering the key role of TNFα in DC biology, the immunomodulatory effects of anti-TNFα therapy on DC function were rapidly explored. One of the pillar articles highlighted that TNFα blockade during Mo-DC maturation results in enhanced apoptosis of the cells [46]. In this study, the authors generated Mo-DC by incubating monocytes with GM-CSF and IL-4 for 6 days, and then exposed the cells to bacterial LPS in the presence of infliximab or etanercept. The blockade of TNFα on surviving LPS-matured Mo-DC resulted in the generation of Mo-DC with a semi-mature phenotype and reduced T-cell stimulatory capacities. Importantly, T cells activated by these semi-mature Mo-DC produced enhanced levels of anti-inflammatory IL-10 and IL-4 and lower levels of pro-inflammatory IFNγ. In line with these in-vitro results, DC derived from RA patients treated with an anti-TNFα therapy had a similar phenotype.

The works detailed above illustrate that TNFα blockade has profound effects on Mo-DC function and could explain, at least in part, the effectiveness of anti-TNFα therapy. When RA patients benefit from this type of therapy, some of them fail to maintain their response after initial efficacy. This phenomenon occurs when the patient develops anti-drug antibodies (ADA) with neutralizing properties, leading to a loss of clinical response and a relapse of the disease. Several parameters are involved in the development of ADA, including the quality of the therapeutic product, and more precisely the presence of protein aggregates that stimulate adaptive immune responses [47]. Importantly, these therapeutic antibody aggregates induce the maturation of Mo-DC that acquire the ability to stimulate Th2 responses, favoring, thus, the production of ADA [48,49].

Experiments similar to those described above on Mo-DC with anti-TNFα therapy were also carried out with tocilizumab, a humanized monoclonal antibody that targets the IL-6 receptor α, that is widely used in RA. Surprisingly tocilizumab do not inhibit IL-6 secretion by Mo-DC differentiated by culturing monocytes with GM-CSF and IL-4 and activated with LPS [50]. This counterintuitive result suggests that Mo-DC could remain a significant source of IL-6 in patients treated with tocilizumab, contributing to the inflammation and joint destruction. It could also explain at least partially why only 30% of RA patients treated with tocilizumab display a remission, and why 20% of them do not respond after 24 weeks [51].

Abatacept is another targeted therapy largely used to treat patients with RA. Its mode of action is based on the inhibition of T-cell activation by binding to the costimulatory molecules CD80 and CD86 expressed by antigen-presenting cells such as DC. An interesting effect of abatacept is its capacity to inhibit the T-cell stimulatory capacities of osteoclasts differentiated from Mo-DC in the presence of M-CSF and RANKL [52]. Indeed, these DC-derived osteoclasts have the particularity of expressing MHC class II molecules as well as costimulatory molecules, and of retaining DC functions, such as the ability to stimulate T cells [52]. So, by this double action on DC-derived osteoclasts, abatacept could prevent both osteoclastic bone resorption and aberrant activation of T cells.

Tofacitinib is a targeted synthetic DMARD well known to dampen inflammatory responses in patients in several chronic inflammatory diseases such as psoriasis or RA. Its mode of action relies on the inhibition of the JAK1/JAK3 signaling, resulting in the blockade of pro-inflammatory signals of several cytokines involved in immune-mediated inflammatory diseases. Curiously, this small Jak inhibitor affects the differentiation of Mo-DC and shifts them towards an M1-like macrophage phenotype that produce IL-12 and IL-23. Although tofacitinib seems to limit T-cell responses by decreasing the level of co-stimulatory molecules on Mo-DC, the enhanced expression of IL-12 and IL-23 might potentially favor the activation of Th1 and Th17 cells, central in the pathogenesis of RA. Therefore, the anti-inflammatory clinical efficacy of tofacitinib does not seem to be explained by a direct effect on Mo-DC but could, rather, be mediated by its anti-proliferative effect on CD4^+^ T cells, and its ability to suppress the production of IFNγ and IL-17 by T cells [53].

## 6. Conclusions

Mo-DC generate a growing interest, driven by numerous studies that suggest a key role played by these cells in the initiation and/or maintenance of chronic inflammatory diseases such as RA. The molecular and cellular factors that govern the differentiation of monocytes into Mo-DC still remain a field of exploration that is both fascinating and largely unknown. To date in RA, four main environmental factors have been identified as greatly impacting the differentiation/activation of Mo-DCs: (i) the agonists of the AhR, (ii) the extra-cellular acidosis, (iii) the GM-CSF produced by synovial CD4^+^ T cells, and (iv) the synoviocytes and the synovial fluid. The integration of all these environmental signals by the monocyte that infiltrate the synovial tissue can promote their differentiation into Mo-DC, fueling locally inflammation and participating to joint destruction by favoring IL-17 production and transdifferentiating toward potent osteoclasts. In addition to these environmental factors, there is also the impact of targeted therapies from which the patient benefits. Other environmental signals still need to be identified and the understanding of their mechanisms of action will undoubtedly lead to the development of new therapeutic strategies in the field of RA and other chronic inflammatory contexts, such as cancer.

## Data Availability

Not applicable.
